# Patient satisfaction with conventional versus fully digital obturators fabricated with 3D printing: a randomized crossover trial

**DOI:** 10.1186/s12903-025-06106-y

**Published:** 2025-05-28

**Authors:** Maha M. AboShady, Marwa M. Amer

**Affiliations:** Department of Prosthodontics, Faculty of Dentistry, Tanta University, ElGeish Street, Box 31527, Tanta, Egypt

**Keywords:** Digital obturator, Intraoral scanning, Selective laser melting, 3D printing, Obturator functioning scale

## Abstract

**Background:**

Evidence concerning the performance of entirely 3D printed digital obturators is insufficient. This prospective randomized, single-blind crossover study aimed to evaluate patient satisfaction with conventional obturators and 3D printed obturators.

**Methods:**

Ten patients aged between 45 and 65 years with unilateral hemi-maxillectomy due to oncological surgeries were enrolled in a crossover design. Initial treatment involved a conventional obturator with a heat-cured acrylic base and a cast metal framework GI. Following a two-week washout, patients received a fully digitally designed and fabricated obturator incorporating 3D printed materials GII. Satisfaction was assessed using the validated Obturator Functioning Scale (OFS), and statistical analysis was conducted using the Mann-Whitney U test.

**Results:**

The median of OFS scores of the two groups were statistically significant differences between the two studied groups in terms of functional and aesthetic limitations, with *p*-values 0.010* and 0.014* respectively. On the other hand, there were no significant differences in chewing limitation and social disability, with *p*-values of 0.572 and 0.087 respectively.

**Conclusion:**

Within the limitations of this study, a full digital workflow for producing maxillary obturator prostheses can increase patient satisfaction with respect to functional, aesthetic, and phonetic outcomes.

**Trial registration:**

This research was registered on www.clinicaltrials.gov in 17-06-2023 with registration number NCT0592081.

**Supplementary Information:**

The online version contains supplementary material available at 10.1186/s12903-025-06106-y.

## Background

The main objective of prosthetic rehabilitation for maxillary defects is to close oronasal connection to prevent fluid leaking into the nose, and hypernasal speech, and enhance functions such as mastication, swallowing, speech clarity and articulation. It also aims to restore facial appearance and enhance both quality of life and patient satisfaction [1, 2]. However, the need to preserve the remaining structures, ensure retention and stability, and overcome functional stresses makes maxillary defects rehabilitation challenging [3–4].

Although conventional maxillary obturator prostheses constructed from a metal framework using (the lost wax casting technique), heat-cured acrylic resin can restore oral function and aesthetics after maxillectomy. However, this method requires significant time and labor. Numerous potential errors can arise during the laboratory processes, leading to an improper fit of the framework [5].

The application of digital technologies has gradually been introduced in the field of maxillofacial prostheses to overcome the inherent drawbacks of conventional methods. Using an intraoral scanner (IOS) can help such patients to overcome the unpleasant experience of conventional impressions. Additionally, there is no chance of any impression material inadvertently becoming lodged in the airway [5–8].

The use of CAD-CAM technologies in the fabrication of maxillofacial prostheses has been described [9–12]. Subtractive manufacturing produces accurate prostheses but has certain disadvantages such as large production machines, high time consumption, high cost, limited block size, and the necessity to dispose of cutting waste [13],. These drawbacks can be overcome if denture construction by additive manufacturing becomes feasible. Compared with subtractive techniques, Additive manufacturing provides a more convenient and effective method with less material waste [14].

Williams et al. [15] were the first to create a 3D printed cobalt-chromium removable partial denture framework via selective laser melting (SLM) technology. SLM is a process that fuses metallic powder together via high-energy laser beams to build 3D objects layer by layer. This process eliminates many physical manufacturing steps such as spruing, investing, burnout, casting and devesting. Therefore, it is faster and less labor-intensive than investment casting, with RPD frameworks potentially being produced in approximately 30 min [16].

Obturator performance can be assessed both objectively and subjectively. Objective evaluation requires the operator’s assessment using scientific equipment, whereas subjective assessments focus on the patient’s perspective toward the prosthesis through questionnaires [17, 18]. Subjective outcomes are particularly important for considering optional treatments as they are often more sensitive in detecting differences between treatments. Hence, patient satisfaction and oral health-related quality-of-life measures are frequently used in clinical trials to assess prosthetic treatment outcomes [17, 18].

The Obturator Functioning Scale (OFS) was developed at Memorial Sloan Kettering Cancer Center (New York, NY, USA) to evaluate the self-reported functioning of an obturator. This 15-item scale designed by Kornblith et al. [19] assesses aspects like eating ability, speech, and cosmetic satisfaction, using a 5-point Likert scale where higher scores indicate greater difficulty with the obturator function. An item regarding ‘‘difficulty talking on the phone’ was included to assess communication challenges without visual cues.

Clinical reports have used combined conventional and digital methods for the obturator prostheses [10, 20, 21, 22]. Clinical research on fully digital workflow for fabricating obturators is deficient. So, the aim of this clinical trial was to assess patient satisfaction between the total digitally fabricated obturator and the conventional obturator. The null hypothesis is that there was no significant difference in patient satisfaction between conventional and fully digitally fabricated obturators.

## Methods

This prospective crossover study was approved by the Research Ethics Committee of the Faculty of Dentistry, Tanta University #R-RP-1-24-3039, and the research was registered on clinicaltrials.gov with registration number NCT05920811. Each participant signed an informed consent form after clarification of the study’s purpose, methods, and participants’ rights. All procedures in this study adhere to declaration of Helsinki.

### Eligibility criteria

The inclusion criteria in the study included patients who underwent hemimaxillectomy (Aramany class 1) at least 2 years before the beginning of the study, with healthy remaining mandibular teeth, had a mouth opening of at least 25 mm, intact soft palate, and participants who had not received radiation therapy or chemotherapy in the preceding year. The exclusion criteria were patients with physical or mental illnesses.

### Patient allocation

A crossover clinical trial was performed on ten partially edentulous adult patients with hemimaxillectomy. The patients were recruited from the outpatient clinic of the Prosthodontic Department, Tanta University. The sample size calculation based on the results of a previous study [23] revealed a sample size of *N* = 10 after calculating the dropout rate (0.2%) to detect a correlation at a significance level of a = 0.05 (*p* < 0.05), the confidence interval 95% and the actual power was 96.97% and the effect size = 2.06721. The sample size was calculated using a computer program G power version 3.1.9.

**Fig. 1:**
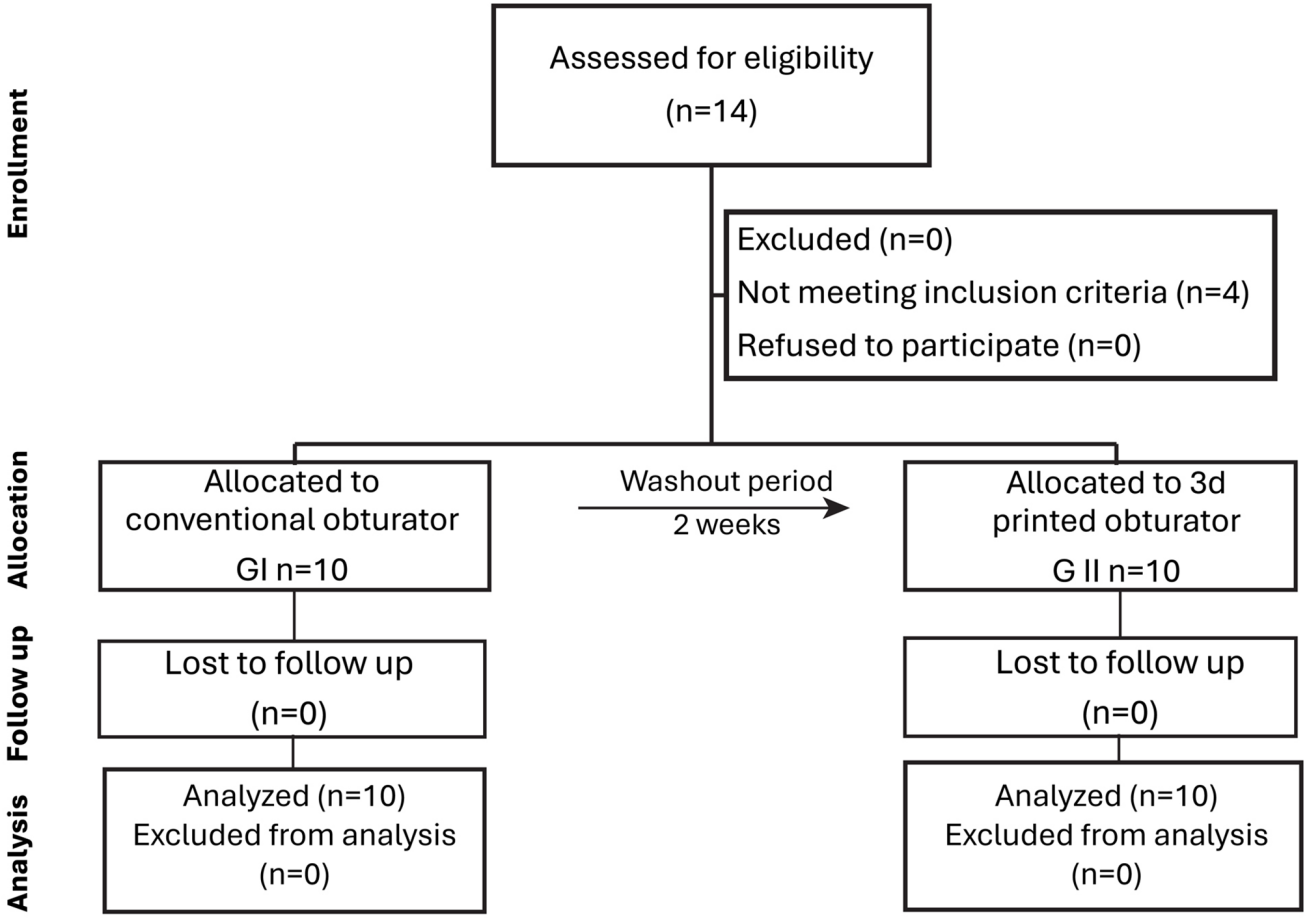
CONSORT flow diagram

All patients received conventional obturator (Group I) for 6 months, after a washout period of two weeks, they used the digital one (Group II) (Fig. 1). All the treatments were performed by the same operator and technician. The patients were blind to the type of prosthesis they would receive because the clinical steps of both obturators (starting from impression and scan to try in) were completed simultaneously.

#### Clinical workflow

Conventional obturators were produced via the traditional steps of obturator construction. After evaluation of the remaining teeth, Primary impressions were made for the maxillary and mandibular arches and poured into dental stone (Type 4 X-hard Stone, Zhermack S.p.A., Italy) to obtain the study cast. The required preparations were performed according to the proposed design for each case including rest seats preparations, guiding planes and occlusal reduction of over-erupted teeth. The final impression was taken via a medium body rubber base (Zhermack Thixoflex M, Zhermack S.p.A., Rovigo, Italy) in a custom tray. The metal framework was designed and fabricated using the lost wax technique and try-in was performed, Bite was recorded. The artificial teeth were arranged, and the wax-up was completed and evaluated intraorally. Then, the definitive obturator was fabricated using heat-cured acrylic resin. The obturator was finished and polished, delivery and final occlusal adjustments were performed intraorally, and then the patient returned for follow-up one week later.

**Fig. 2:**
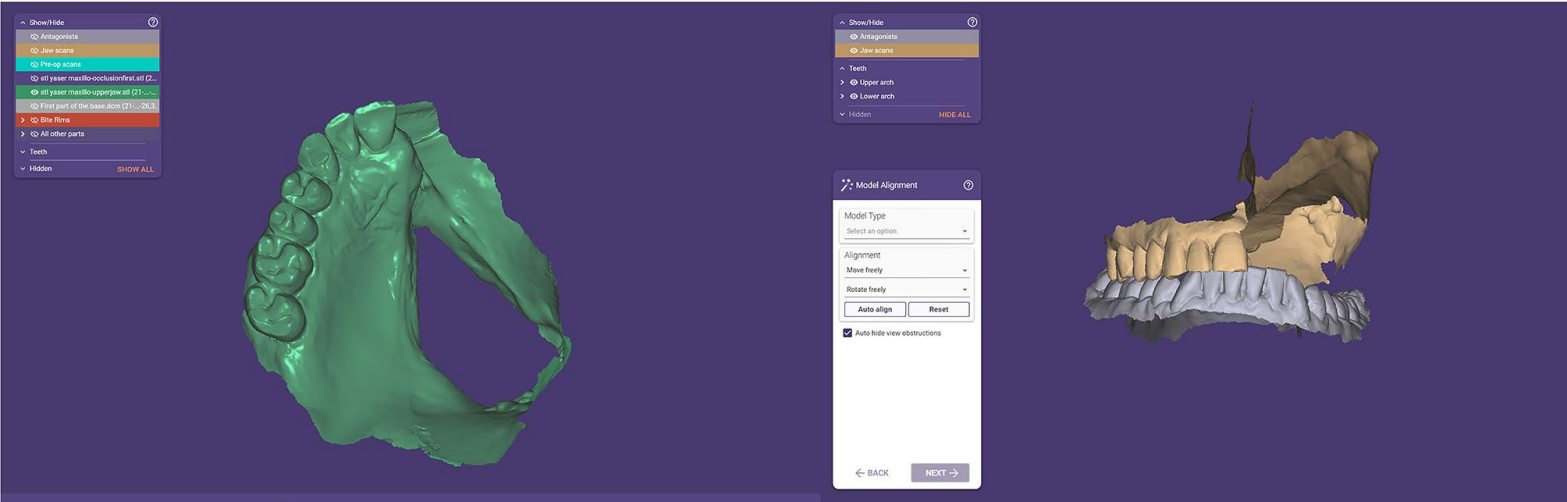
Intraoral scan of the maxillary arch with the defect and scanning of the bite

**Fig. 3:**
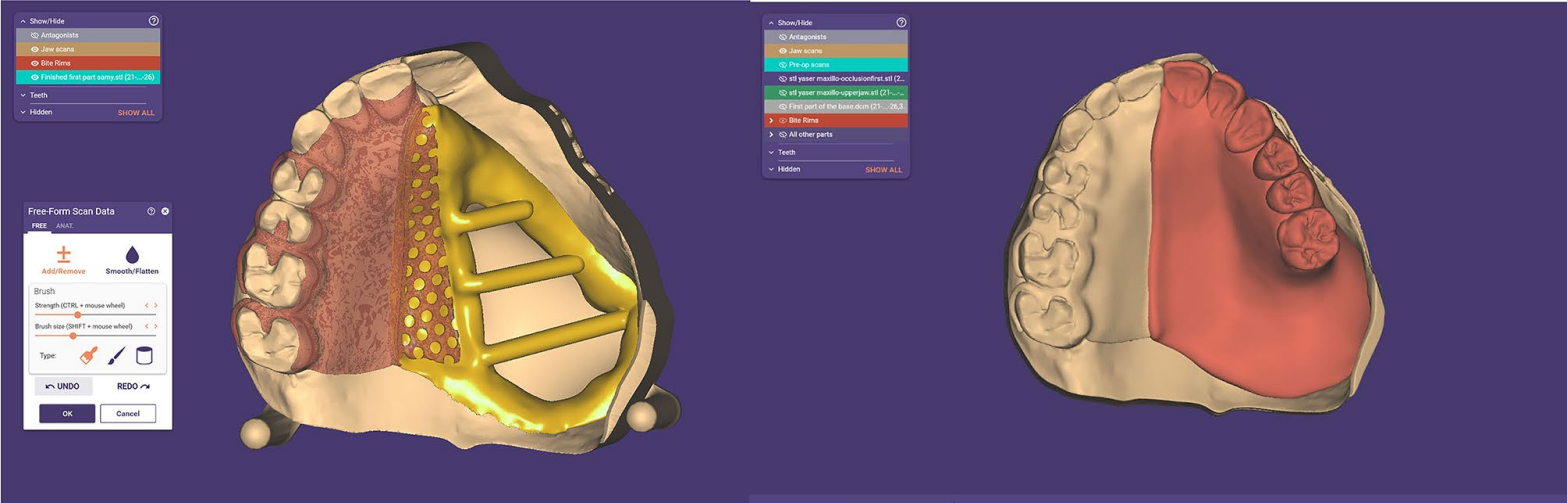
Digital design of the obturator parts

**Fig. 4:**
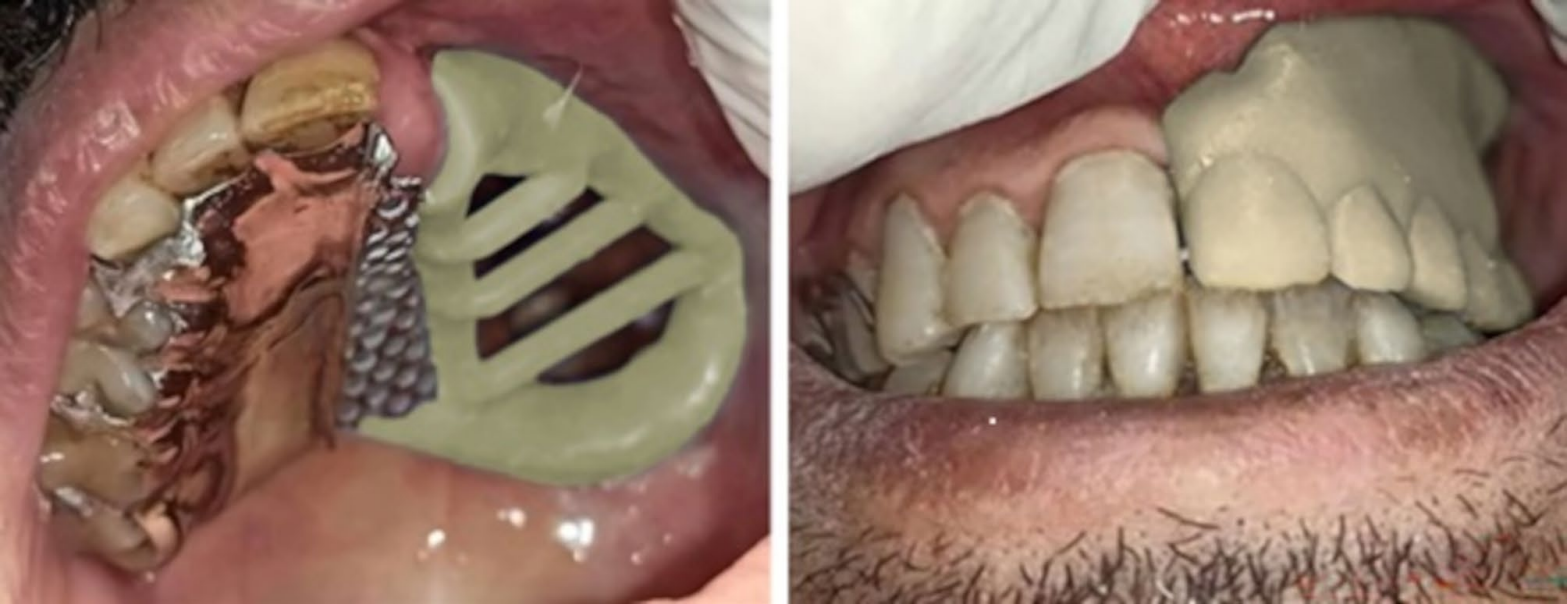
Try in of the metal framework with the acrylic parts of the digital obturator

For digital obturators, a complete digital workflow was followed starting with scanning of both arches including the defect, teeth and occlusion with an intraoral scanner (Medit i500, Korea) (Fig. 2). The scan strategy for the maxillary arch started from the natural teeth passing through the palate and finally scanning the defect. The patients were instructed to remain stable without movement while the cheek retractor was kept steady. The scanned data was imported into a CAD software program (exocad DentalCAD 3.0 Galeway). Virtual surveying and virtual blocking of the unwanted undercuts of the teeth and the cavity follow the same design of the conventional framework. A meshwork minor connector was added at the midline extending to the defect for the attachment of the denture base part, and then the designed framework was exported as STL file to be imported into SLM machine (MetalSys 250, WinforSys, Yongin, Korea), The metal framework was 3D printed in cobalt-chrome (Co-Cr) powder (EOS CobaltChrome, EOS GmbH, Germany). After 3D printing, the support structures were cut, and the framework was finished and polished following the same procedures used for conventionally manufactured frameworks. The acrylic part was designed into two parts, the tissue part of the obturator prosthesis that covers the boundaries of the defect and engages 2 mm depth and the polished part that carries the teeth and forms the polished surface of the obturator. The two parts were designed and 3D printed in (NORTON premium resin, China) using Anycubic 3D printer (Anycubic Photon S - High-precision SLA 3D Resin, Shenzhen, China) for try-in with the metal framework (Fig. 3). The maxillary model was printed for evaluation of the fit of the metal framework and the acrylic parts, then they were evaluated in the patient’s mouth for retention, adaptation and occlusion (Fig. 4). The final acrylic parts were 3D printed in tooth-colored resin material (NextDent C&B MFH, MicroFilled Hybrid, Netherlands). All parts were positioned over the master cast to be bonded. The metal primer (MKZ Primer, bredent GmbH & Co, Senden, Germany) was applied to the mesh part of the metal framework, and the three parts were bonded with the same light-cured resin used for 3D printing of the final acrylic parts, Pink visiolign (crea.lign paste GUM PC40, bredent GmbH & Co, Senden, Germany) was added to the polished surface of the resin base to simulate gingival color. Intraoral insertion was performed, and the prosthesis was evaluated for aesthetics, retention, comfort, speech and swallowing.

### Evaluation

A translated form of the Obturator Functioning Scale in the Arabic language was completed by the patients in the absence of prosthodontists. The questionnaires were recorded three months after each prosthesis insertion. All questionnaires were conducted by the same research interviewer, who was blinded to the type of obturator.

### Statistical analysis

The data obtained were evaluated using (The IBM SPSS Statistics version 26). Medians and interquartile range (IQR) were used to describe the data, and the Mann Whitney U test was used to assess the differences between the two groups. Not all data had a normal distribution according to the Shapiro-Wilk test of normality.

## Results

The median and IQR of Functional Obturator Scale scores and for each question, subscale, and total score between the two groups are shown in Table (1) and (2). A *P*-value of less than 0.05 (*) was considered to indicate a significant difference.

**Fig. 5:**
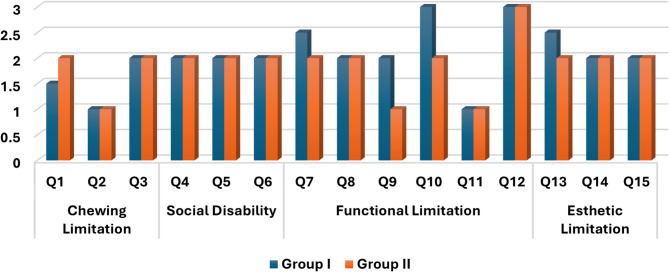
Differences in the median OFS score between the two groups at each question

The conventional group showed a statistically significant increased score as compared with the digital group with respect to difficulties in inserting the obturator (*p* = 0.046*), difficulty in understanding the speech (*p* = 0.008*), Difficulty in talking on the phone (0.005*), and Funny looking of the upper lip (*p* = 0.012*). On the other hand, there were nonsignificant differences between both groups with regard to the remaining parameters (Table 1), (Fig. 5).

**Table 1:** Median and interquartile range of OFS scores between the two groups of each question

Question	Group I	Group II	Mann Whitney U -test
	Median (IQR)	Median (IQR)	Z (*p*-value)
**Chewing Limitation**			
Difficulty in chewing food	1.50 (1)	2 (2)	0.938(0.348)
Xerostomia (dry mouth)	1 (1)	1 (1)	0.775(0.439)
Leakage during swallowing food	2 (2)	2 (2)	0.744(0.457)
**Social Disability**			
Patients avoid going in family and social events and functions	2 (1)	2 (1)	1.582(0.114)
Numbness in the upper lip	2 (2)	2 (1)	0.491(0.623)
Faces difficulties while inserting an obturator	2 (1)	2 (1)	1.991(0.046*)
**Functional Limitation**			
Difficulty during talking in public	2.5 (1)	2 (1)	1.575(0.115)
Difficulty in pronouncing different words	2 (1)	2 (1)	1.424(0.154)
Speech difficult to understand	2 (1)	1 (1)	2.646(0.008*)
Difficulty in talking on the phone	3 (1)	2 (1)	2.813(0.005*)
Nasal speech	1 (1)	1 (1)	0.639(0.523)
Change of voice before and after the surgery	3 (1)	3 (1)	0.681(0.496)
**Esthetic Limitation**			
Funny looking of the upper lip	2.5 (1)	2 (1)	2.514(0.012*)
Weird look or dissatisfaction with look	2 (0)	2 (1)	1.760(0.078)
The noticeable clasp of obturator on front teeth	2 (0)	2 (0)	0.485(0.628)

Data was expressed using Median and IQR

*: Statistically significant at *p* ≤ 0.05

**Table 2:** Median and interquartile range of OFS between the two groups at each scale

Variable	Group I	Group II	Mann Whitney U -test
	Median (IQR)	Median (IQR)	Z (*p*-value)
Chewing Limitation	1.67 (1.08)	2 (1)	0.565(0.572)
Social Disability	2.17 (1.08)	2 (0.42)	1.713(0.087)
Functional Limitation	2.42 (0.05)	1.83 (0.71)	2.575(0.010*)
Esthetic Limitation	2.17 (0.42)	1.83 (0.75)	2.458(0.014*)
**Total**	**2.27 (0.47)**	**1.73 (0.53)**	2.616(0.009*)

The differences in the medians and IQR of OFS between the two groups at each scale were shown in Table (2). There were statistically significant differences between the two studied groups in terms of functional and aesthetic limitations with *p*-values 0.010* and 0.014* respectively. On the other hand, there were non-significant differences between the two studied groups in terms of chewing limitation and social disability, with *p*-values of 0.572 and 0.087 respectively. The median total FOS scores for Group I and Group II were 2.27 and 1.73 respectively, which were significantly different with *p*-values 0.009*.

## Discussion

The patient’s oral functions and aesthetics can be restored with a properly designed definitive prosthesis with a framework that can gain retention, support and stability from the remaining teeth and a properly adapted base with maximal coverage, and proper extension. The metal framework gives the prosthesis the advantages of longevity, thermal conductivity and superior mechanical properties which result in increased resistance to deformation and efficient transfer of occlusal forces to the remaining teeth or other tissues [24]. These characteristics are also crucial for obtaining a tissue-compatible satin finish which is attained through electrolytic polishing to avoid fitting and cleaning challenges related to the fitting surface of Co-Cr frameworks [25].

Achieving proper retention and optimal stability of obturator protheses via conventional techniques presents a significant challenge for prosthodontists, as multiple impressions are needed to construct diagnostic, master, and altered casts, which may be uncomfortable for patients with maxillary defects [26, 27]. Conventional impressions taken for obturators in maxillectomy patients also have a substantial risk of aspiration, foreign body entrapment, and impression deformation during removal from large undercuts [28, 29].

The use of CAD/CAM technology in dentistry has grown to allow the creation of intraoral digital impressions, providing high-resolution data for both arches and the interocclusal bite directly with less labor and faster results than conventional method does. Additionally, virtual surveying of the model and virtual design of the obturator facilitates the determination of the desirable undercuts, and guiding planes, allowing for an optimal path of insertion, and controlling the thickness and extension of the base and framework without requiring a dental surveyor, duplicate impressions or refractory casts, so it offers reduced laboratory time and preserves data for future prosthesis reproduction [30].

SLM has the advantage of fabricating removable partial dentures with sufficient accuracy and rigidity. Compared with milling manufacturing, it enables the printing of complex designs with acceptable clinical fit of denture frameworks and reduces waste material. Previous studies have shown that SLM-produced Co-Cr alloys exhibit sufficient mechanical properties for clinical use and are superior to cast Co-Cr alloys [31–34]. Cytotoxicity tests have also proved that SLM-produced Co-Cr alloy is safe, non-irritating and non-toxic to oral tissues and the body overall [35, 36].

In this study, the acrylic part of the obturator was designed to be roofless to decrease the weight of the prosthesis and allow easy cleaning. The digital acrylic part was designed into two separate parts, to allow the metal to be sandwiched between them. Future debonding of the teeth from the denture base was prevented by 3D printing them in one part with the polished acrylic part, and then pink visiolign was added to the polished surface for aesthetics.

The findings of this study revealed significantly higher scores in the conventional group regarding the funny appearance of the upper lip, difficulty in inserting the obturator, difficulty in understanding speech and difficulty in talking on the phone. These results may be attributed to the increased bulk of the acrylic part of the conventional obturator. This may be caused by the impression material that raises the upper lip with funny looking. Additionally, the pressure of the impression material in the defect while taking the impression results in a thicker acrylic part of the conventional obturator causing it to fit deeper into the defect especially from the medial side leading to difficulties in obturator insertion. Furthermore, virtual surveying allows easier and more accurate surveying than manual surveying does, facilitating easier insertion and removal of the prosthesis [37].

The digital scanning and design enable accurate fit of the digital prosthesis. The improved mechanical properties of the 3D printed metal framework with its accurate fit, lead to increased patient satisfaction with speaking, mastication, and comfort. These findings agreed with those of Soltanzadeh et al. [5], who reported that rapid prototyping could achieve a clinically acceptable fit for metal frameworks. Additionally, Jiang et al. [38] and Jiao et al. [39] reported that most patients felt more comfortable with their facial appearance after wearing the digital obturator and that their speech ability improved after insertion. Juanita et al. [27] indicated that most patients using digitally fabricated palatal obturator had minimal problems with eating, speech, and other functional items. Park et al. [26] and Tasopoulos et al. [10] found that the use of the CAD/CAM prosthesis improved the quality of life of patients and achieved more precise repair after total maxillectomy, based on evaluations of the facial profiles and functional recovery of postoperative patients. Brucoli et al. [7] revealed that when the maxillary obturator was made using an optical scanner, 93% of patients claimed that there was no fluid leakage, most patients also reported a good or complete recovery of phonation and swallowing.

The results revealed a significant difference between the median total OFS of conventional and digital groups, so the null hypothesis was rejected.

The limitations of this study include the short term follow up and the relatively small sample size. Future clinical research with a larger sample size with more diverse patient populations should better analyze the potential difference between digital and conventional obturators. Also, the design of the obturator prosthesis itself may influence patient experiences; other designs such as hollow bulb obturator should be investigated.

## Conclusion

Within the limitations of the present study, a full digital workflow for producing maxillary obturator prostheses can increase patient satisfaction in terms of functional, aesthetic, and phonetic outcomes.

### Recommendations

The long-term evaluation of digitally fabricated maxillary obturators with different Aramany classes and different materials requires future investigation.

## Electronic supplementary material

Below is the link to the electronic supplementary material.

**Supplementary Material 1**: Consort 2010 check list was uploaded on the system.

## Data Availability

The datasets used and/or analyzed during the current study are available from the corresponding author upon reasonable request.

## Abbreviations

CAD: Computer-aided design
CAM: Computer-aided manufacture
IOSs: Intraoral scanners
STL: Surface tessellation language
SLM: Selective laser melting
3D: 3-Dimensional
OFS: Obturator functioning scale
IQR: Interquartile range
